# Alveolar Ridge Preservation with Autologous Platelet-Rich Fibrin (PRF): Case Reports and the Rationale

**DOI:** 10.3390/dj11100244

**Published:** 2023-10-23

**Authors:** Cemal Ucer, Rabia S. Khan

**Affiliations:** I.C.E Postgraduate Dental Institute, University of Salford, Manchester M5 4WT, UK; ucer@icedental.institute

**Keywords:** platelet-rich fibrin, ridge augmentation, socket augmentation, grafting, cytokines, growth factors, tissue regeneration, bio-enhancement, PRF, platelet concentrate, accelerated implant treatment

## Abstract

In dental implantology, alveolar ridge preservation (ARP) has emerged as a standard technique to address dimensional changes that affect alveolar ridge morphology following tooth loss. Various alternative graft materials, including xenografts, alloplasts, and allografts, have been effectively employed in fresh extraction sites for ARP. Current evidence suggests that these materials primarily serve as bio-scaffolds, which are slowly incorporated, thus necessitating a waiting period of at least 4–6 months before implant placement. Consequently, the ARP technique extends the overall duration of implant treatment by several months. Recently, the incorporation of a form of autologous platelet concentrate, known as platelet-rich fibrin (PRF), has been advocated in conjunction with ARP as a method of bioenhancement of soft- and hard-tissue healing and regeneration. PRF contains platelet-derived growth factors, hormones, and bioactive components like cytokines that have demonstrated the ability to stimulate angiogenesis and tissue regeneration throughout all phases of wound healing. Additionally, the concentration of leukocytes present in the PRF matrix plays a vital role in tissue healing and regeneration as part of the osteoimmune response. The reported advantages of incorporating autogenous PRF platelet concentrates during ARP encompass reduced healing time, improved angiogenesis and bone regeneration, socket sealing through the fibrin matrix, antibacterial properties, and decreased post-extraction pain and infection risk. Therefore, the objective of this paper is to review the existing evidence regarding the application of PRF in alveolar ridge preservation (ARP) following tooth extraction. Two clinical case studies are presented, wherein ARP was enhanced with PRF, followed by implant placement within a relatively short period of 8 weeks. These cases serve as further proof of concept for supporting the adjuvant use of PRF to enhance healing and accelerate implant placement after ARP.

## 1. Introduction

### 1.1. Use of Platelet-Rich Fibrin (PRF) in Dentistry

Autologous platelet concentrates have been employed in dentistry for several years to promote both hard- and soft-tissue healing. Numerous clinical studies, including randomized clinical trials (RCTs), have highlighted the benefits and bioactive role of platelet concentrates in angiogenesis, cell recruitment, differentiation, mineralization, and tissue regeneration during wound healing. Since Marx et al. introduced the application of platelet-rich plasma (PRP) in oral defects in 1998, this technology has gained increasing popularity in oral surgery and implantology. This trend has been especially notable with the introduction of new, less complex, and more effective second and third-generation platelet concentrate preparation protocols in recent years [1].

A recent systematic review conducted by Yu et al. in 2022 revealed a significant surge in annual publications on PRF, encompassing original research articles (58.01%), reviews (17.08%), and case reports (10.14%). In the field of dentistry, PRF has been frequently utilized in various applications, including oral surgery (31.14%), periodontal regeneration (22.42%), and implant therapy (18.68%) [1,2]. The primary purpose of using PRF is to facilitate natural wound healing and enhance tissue regeneration, leading to improved clinical outcomes. In the field of oral surgery, the reported benefits of PRF therapy include reduced morbidity and pain, accelerated wound healing, and the regeneration of new bone and soft tissue following tissue injury. The fundamental mechanism through which platelet concentrates expedite wound healing lies in their capacity to provide substantially higher concentrations (6–8 times higher) of platelet-derived substances, including growth factors, cytokines, and immune system signaling molecules. These substances play a pivotal role in stimulating the recruitment, differentiation, and activation of mesenchymal stem cells, ultimately leading to enhanced tissue healing and regeneration [3].

While the original protocol by Marx et al. (1998) involved the addition of bovine thrombin to obtain PRP [4], Anitua introduced a protocol for producing platelet-rich plasma (PRP) concentrate using CaCl and heat treatment, which he termed “platelet-rich growth factors” (PRGF) [5,6]. These products excluded leukocytes from the fibrin matrix, and the anticoagulant additive used in preparing PRP was later found to be detrimental to tissue regeneration [3,4]. A simpler second-generation plasma concentrate protocol (PRF) was subsequently introduced by Choukroun, involving the creation of a fibrin clot through a novel centrifuge spinning technique. This streamlined method does not necessitate the addition of an anticoagulant or chemicals to the autologous blood sample [7,8,9,10].

### 1.2. Improved PRF Protocols-A-PRF, A-PRF+ and i-PRF

Depending on individual surgical requirements, PRF can be prepared in solid and/or liquid form (e.g., A-PRF, i-PRF, A-PRF+) by altering the G-force during centrifugation (Table 1 and Table 2). Both matrices are rich in growth factors and the differences between different second-generation platelet concentrates have been shown to be small and probably clinically not significant [11,12]. The second-generation PRF concentrates such as advanced platelet-rich fibrin (A-PRF), A-PRF+ and injectable PRF (i-PRF) use lower centrifugal “g-forces” to obtain higher growth factor release compared with the original PRF protocol. The slower centrifugation reduces cell loss and increases the concentration of leukocytes in the PRF matrix (mostly in the buffy coat layer). The effect is to increase the concentration of the total number of cells (neutrophils, lymphocytes, undifferentiated monocytes, and immune cells) as well as the growth factors that are actively involved in bone and soft-tissue regeneration. Advanced PRF (A-PRF) is obtained using reduced g-forces; at 1500 rpm (230 g) for 14 min or at 1300 rpm (200 g) for 14 min [11] (see Table 1).

**Figure 1:**
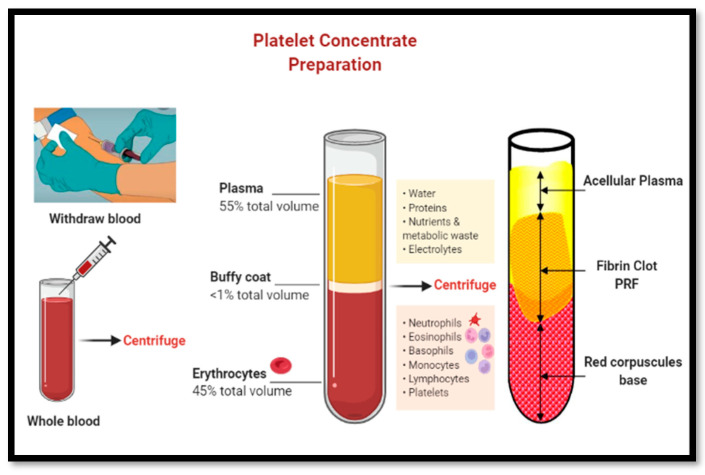
PRF protocol: distinct stages from blood drawing, centrifugation, and clotting process. The buffy coat is a thin layer of highly concentrated leukocytes and platelets at the “face” of the PRF matrix bordering the RBC zone below. Studies have shown a significantly higher release of GFs and cytokines from the cells in the buffy coat layer.

The centrifugation protocols vary between different centrifuge devices and can be calculated using a formula based on the radius of the rotor arm of the device and the speed of rotation (refer to Table 2). Generally, slower centrifugation protocols of 1300 rpm × 8 min produce a more evenly distributed number of platelets compared to the original PRF protocol of 2700 rpm × 14 min. Furthermore, Kobayashi et al. (2016) [13] have demonstrated a much-sustained release of GFs from slower PRF preparations (e.g., A-PRF) compared to the original PRP or PRF protocols [13,14]. The injectable-PRF (i-PRF) protocol (60 g g-force at 3 min) produces the highest concentration of leukocytes/platelets in a smaller volume of liquid matrix. Plastic (PET) tubes used in the i-PRF protocol do not activate the coagulation process since they have hydrophobic non-coated surfaces (see Figure 1). Therefore, a liquid plasma concentrate rich in growth factors, platelets, and bioactive substances forms quickly at the top of the tube [13,14,15].

**Figure 2:**
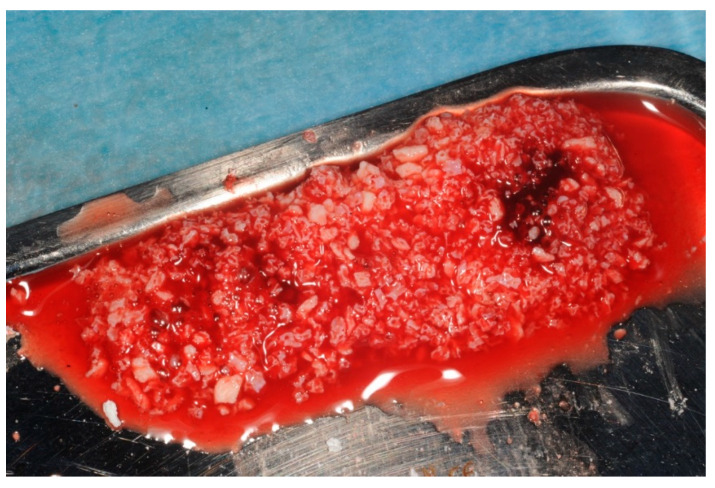
“Sticky bone” is produced by mixing PRF liquid fraction with a bone-substitute material (BioOss^®^ Geistlich).

The buffy coat layer, at the interface, contains a highly dense layer of leukocytes or white blood cells (WBC). When collecting the PRF clot, the buffy coat face should be carefully separated from the red zone of RBC to preserve the WBC layer [14,15]. The solid fibrin matrix (PRF membrane) can be sutured or applied as a wound dressing in conjunction with the guided bone regeneration (GBR) technique or can be packed into a fresh extraction site (fibrin plug) or a large bony defect to enhance tissue repair as part of ARP. PRF membranes remain solid and release large quantities of GFs for 7 to 14 days. Liquid PRF can be mixed with a bone-substitute material (e.g., xenograft or allograft) to produce a coagulated mixture known as “sticky bone” (see Figure 2). This mixture acts not only as a physical scaffold to support new bone formation but also releases bioactive substances such as cytokines and growth factors (e.g., VEGF, PDGF, and TGF) to enhance tissue regeneration (refer to Table 3).

More recently, Miron et al. (2020) [10] managed to further increase the platelet and leukocyte concentration by over 10-fold using a new method of harvesting platelet-rich fibrin (C-PRF) from the buffy coat portion of the platelet concentrate. Tunali et al. (2014) described the titanium-prepared platelet-rich fibrin protocol (T-PRF) using an identical centrifugal protocol to A-PRF but using titanium tubes, arguing that titanium would help activate platelets more effectively [16]. However, the clinical benefit of this technique remains to be demonstrated.

PRF has been successfully used in conjunction with socket augmentation, alveolar ridge grafting, GBR procedures, maxillary sinus grafting, treatment of periodontal and peri-implantitis defects, and soft-tissue grafting in dentistry. Although a PRF membrane does not act as a true barrier membrane, it contributes directly to repair and regeneration at all stages of wound healing. Clinical and histomorphometric research has shown significantly more bone regeneration within bone defects when PRF is combined with xenografts (demineralized bovine matrix/Bioss^®^) or allografts (demineralized freeze-dried bone) [17,18,19]. The reported benefits also include no need for primary wound closure, less ridge resorption, and improved bone quality histologically and density radiologically, with a lower incidence of pain and alveolitis [20].

## 2. Methodology

The objective of this paper is twofold: first, to demonstrate the use of PRF in conjunction with ARP and to investigate whether it can potentially shorten the overall duration of implant rehabilitation following tooth extractions; second, to provide a narrative review of the current evidence supporting the bioenhancement role of PRF when applied alongside alveolar ridge preservation (ARP).

A literature search was conducted using the keywords PRF, ARP, bioenhancement, and accelerated implant treatment after tooth loss, with the inclusion criteria limited to randomized clinical trials (RCTs) only. The search results did not permit a systematic review due to the lack of structured study designs or consistent outcome measures across the RCTs. Most importantly, the papers encompassed various PRF preparation methods and the use of a variety of biomaterials in ARP. Therefore, in this paper, the authors have opted to conduct a comprehensive narrative review and critical analysis of the current evidence available for ARP with PRF. Additionally, two clinical case studies are presented as proof of concept.

### 2.1. Materials and Methods

To demonstrate the concept of accelerated implant treatment following ARP and PRF treatment, two case studies are included. The case studies were selected from patients attending our center requiring tooth extraction and ARP when immediate implant placements were not feasible or contraindicated according to established criteria (XV European Workshop in Periodontology Consensus).

Study Design:
Step 1: Cases were selected for ARP and PRF according to established selection criteria (Ucer and Khan, 2023) [21]
Step 2: Pre-treatment clinical and radiological assessments were undertaken to determine suitability for ARP
Step 3: Teeth were removed using a minimally invasive extraction technique and the sockets were grafted with a xenograft and PRF
Step 4: ARP graft healing was monitored radiologically. A CBCT was taken to plan for implant placement
Step 5: Implant placement was carried out under LA
Step 6: Implants were loaded early and monitored clinically and radiologically

Both cases were treated by a single oral surgeon (CU) in an outpatient setting under local anesthetic. Clinical and radiographical follow-up assessments were conducted to evaluate the healing process. A comprehensive informed consent process was undertaken after detailed discussions of the proposed treatment with the patients.

### 2.2. Alveolar Ridge Preservation Technique

**Figure 3:**
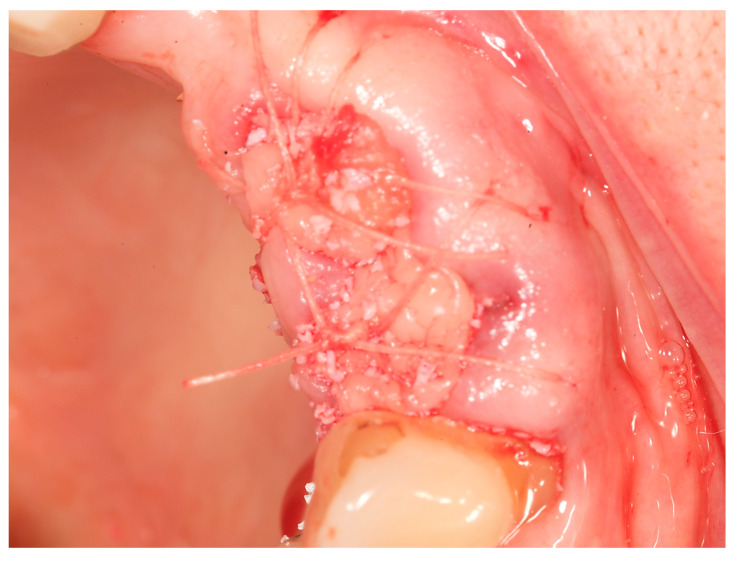

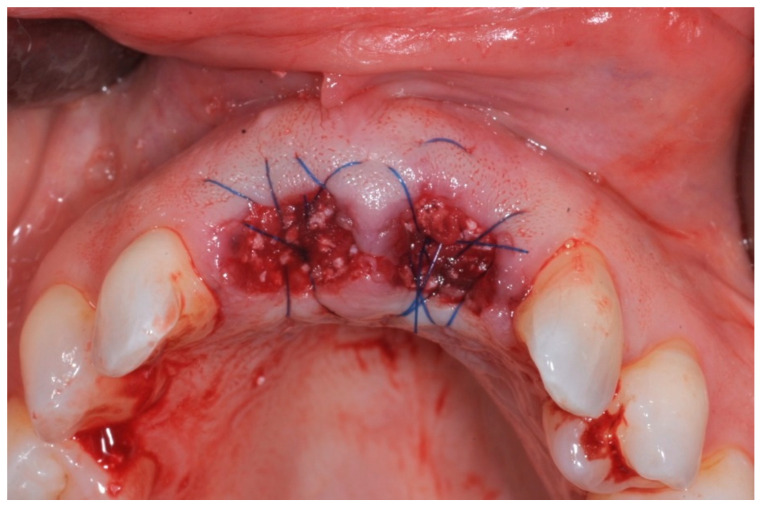
Semi-open minimally invasive alveolar ridge preservation technique with PRF fibrin: tooth extraction was carried out with a periotome to prevent fracture/loss of the thin buccal plate. A soft-tissue flap was not raised to avoid damage to the periosteum and the blood supply. The socket was degranulated and irrigated with saline and grafted with “sticky bone” produced by mixing a bone-substitute material BioOss^®^ Geistlich and i-PRF liquid. The graft was covered with an A-PRF fibrin clot which was sutured to the edges of the socket without any attempt to achieve primary closure. When using a “sticky bone” mixture of PRF and a biomaterial, it may not be essential to cover the extraction site with PRF fibrin membrane although evidence suggests that using 2 layers of PRF over the ARP graft would be highly beneficial.

The alveolar ridge preservation (ARP) procedure adhered to previously published criteria (Ucer and Khan, 2023) [21] after a thorough clinical and radiological evaluation of the tooth and ridge conditions. The initial surgical step involved tooth extraction performed under local anaesthesia (4% articaine with 1:200,000). A minimally invasive extraction technique was employed, avoiding the elevation of a periosteal flap, with careful attention to preventing damage to the socket walls. The sockets were meticulously degranulated to remove any remaining soft-tissue debris. Subsequently, the sockets were immediately grafted using a xenograft (Bioss^®^ small particles) mixed with i-PRF (liquid) to create a coagulum of “sticky bone” (refer to Figure 2 and Table 1). The i-PRF (liquid) concentrate was prepared by collecting 10 mL of venous blood and utilizing a centrifugation protocol at 700 rpm for 3 min (see Table 1). Centrifugation was initiated within 90 s of blood collection, following the recommendation by Miron et al. (2021) [22]. No barrier membranes were employed, and the site was covered with a PRF membrane composed of a fibrin clot, which was sutured to the socket edges using 3.0 Vicryl^®^ Rapide (see Figure 3). There was no flap retraction, and consequently, no primary closure was performed, allowing the sockets to heal semi-openly via secondary intention. PRF fibrin clot was used to cover the soft-tissue defects resulting from the extraction and was stabilized using a mattress suture technique. After an 8-week period, a radiological assessment of the grafted sockets was conducted using CBCT. Implants (Megagen^®^ Anyridge) were placed 1 mm sub-crestally with excellent primary stability (insertion torque > 60 Ncm). These implants were fitted with transmucosal healing abutments (5 mm diameter and collar height of 3 mm) and allowed to heal trans-mucosally before being loaded 6 weeks after placement. Clinical and radiological follow-up examinations at 6 months revealed satisfactory peri-implant soft-tissue parameters with no radiological signs of marginal bone loss.

### 2.3. Outcome Measures

The outcome measures used to assess socket healing, bone conversion, and implant survival included:

(I) Primary outcome measures:
  (a) radiological assessment of bone quality and quantity after ARP using cone beam computerized tomography (CBCT)
  (b) Intra-operative assessment of bone quality, density, and primary implant stability during implant placement surgery
  (c) successful early placement and loading of implants

(II) Secondary outcome measures:
  (a) presence or absence of implant mobility at the time of loading
  (b) need for additional grafting at the time of implantation
  (c) radiological assessment of implant integration and marginal bone integrity after loading.

### 2.4. The Radiological Protocol

The following radiological protocol was used to assess the condition of the extraction sockets and the stability of peri-implant bone after implant placement according to aforementioned outcome measures.

(a) Preoperative assessment of the tooth to be extracted using periapical radiographs
(b) A CBCT scan of the sockets 8 weeks after ARP, before implant placement
(c) Baseline radiographs at the restorative loading stage
(d) Periapical radiographs at 6 months and 12 months after baseline

## 3. Results

Two cases presented in this paper demonstrate the successful rehabilitation of the augmented sites with dental implants just 8 weeks after ARP with PRF.

To serve as a proof of concept, the primary objective of this study was to examine whether PRF could facilitate early implant placement (e.g., at 8 weeks as opposed to 24 weeks) and early implant loading (e.g., 6 weeks after implant placement in ARP sites) when utilized alongside ARP. The results are summarized in Table 4.

### 3.1. Case Study 1

**Figure 4:**
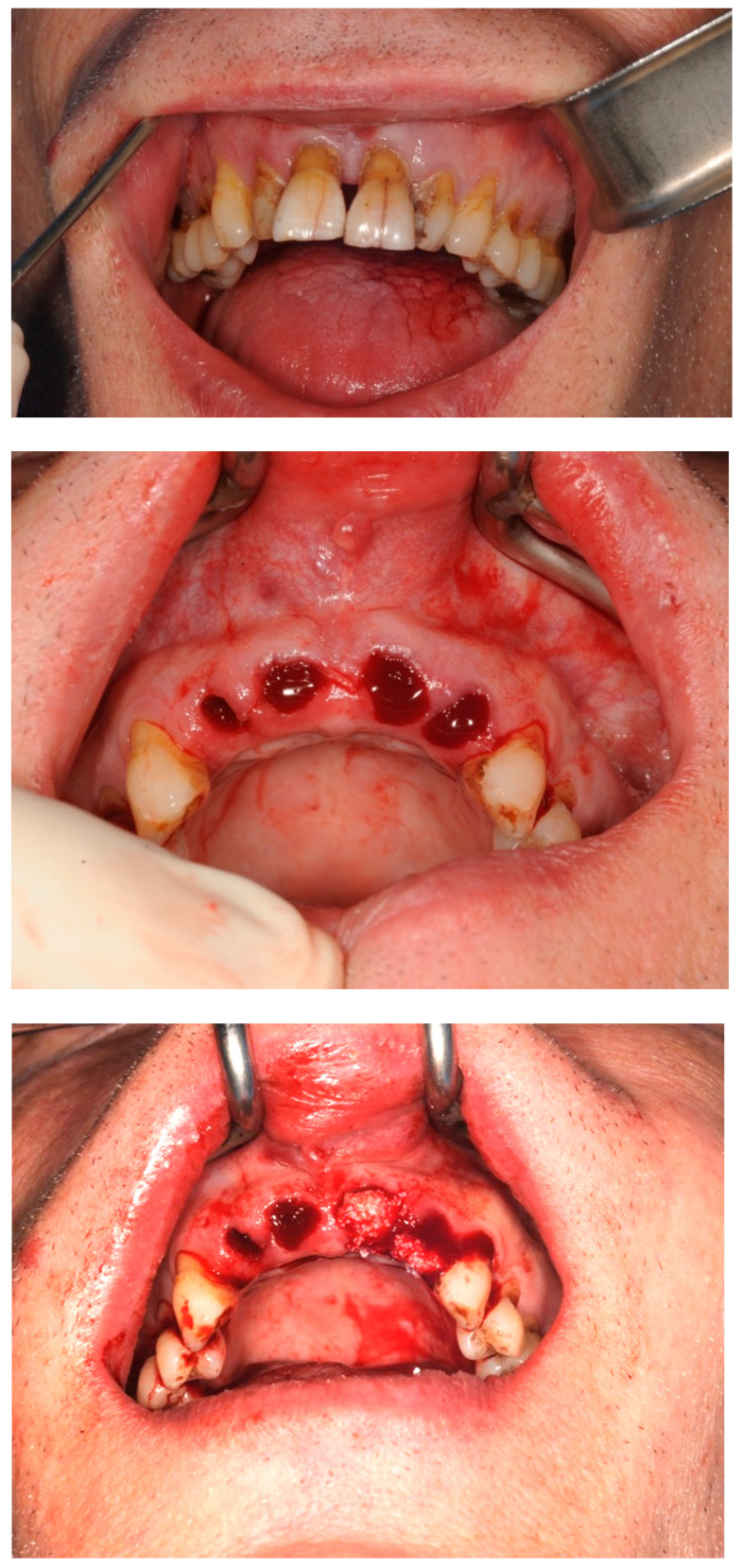

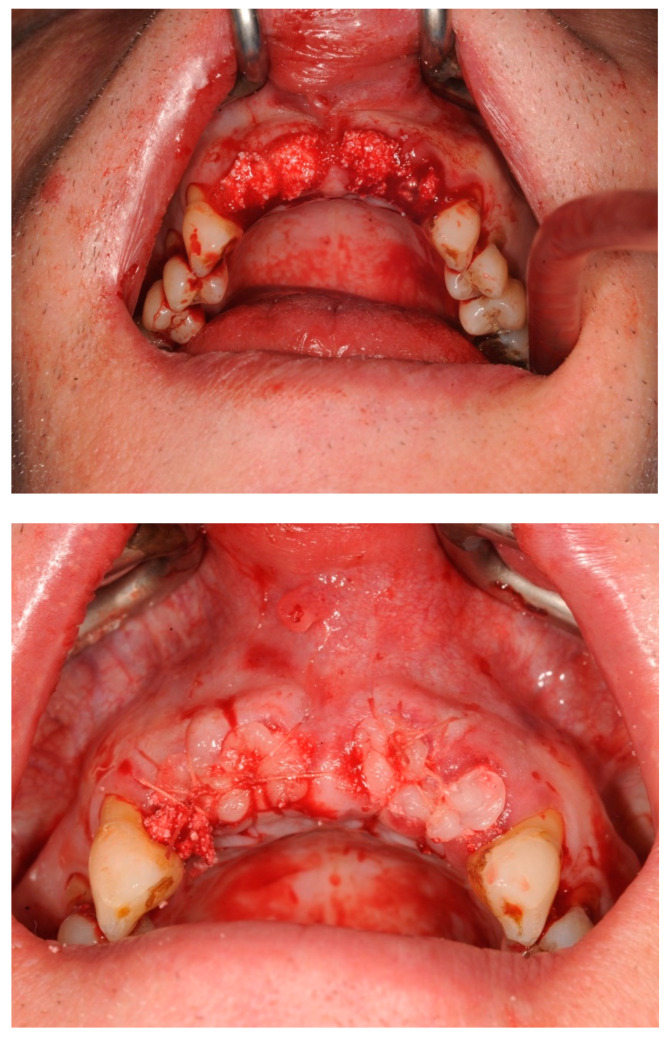
Extraction sockets of periodontally involved upper incisor teeth which were augmented with a xenograft (BioOss ^®^, Geistlich Biomaterials) (Switzerland) mixed with i-PRF and closed with a double layer of A-PRF membrane using Vicryl ^®^ 3.0 sutures.

A 60-year male generally fit and well with a recent history of pre-diabetes and no smoking history presented complaining of the poor aesthetics associated with his anterior teeth. He gave a recurring history of infection from these teeth with the last episode being 6 months earlier. Clinically, his oral hygiene was fair to poor with marginal plaque deposits and probing depths generally up to 4 mm. The upper incisor teeth, however, had probing depths extending to the root end with grade 2/3 mobility and recession (Figure 4). A diagnosis of chronic periodontitis with possible perio-endo lesions on the upper anterior teeth was made. The prognosis of the teeth was assessed to be poor and after a course of initial periodontal treatment and stabilization, the extraction of the upper anterior teeth was planned with interim replacement using an Essix retainer. Due to the labial bone defect, the decision was made to extract the teeth and carry out ARP using a xenograft (Bioss Geistlich^®^) mixed with i-PRF. The extraction defect was treated with a double layer of A-PRF fibrin matrix membrane (produced in accordance with the A-PRF protocol presented in Table 1) and was sutured (Vicryl® 3.0) to the mucosa surrounding the edges of the extraction sockets (Figure 4).

Four maxillary incisor teeth were extracted using the minimally invasive extraction technique according to the protocol described above, and the remnants of any granulation tissue were removed. The extraction sockets were augmented using a xenograft (Bio-Oss^®^/Geistlich Biomaterials) mixed with i-PRF (see Table 1). Two Megagen Anyridge^®^ implants were subsequently placed and restored with a 4-unit bridge after successful control of the periodontal disease (see Table 4). Good incorporation of the particulate xenograft surrounded by newly formed bone was noted at the time of implant placement.

**Figure 5:**
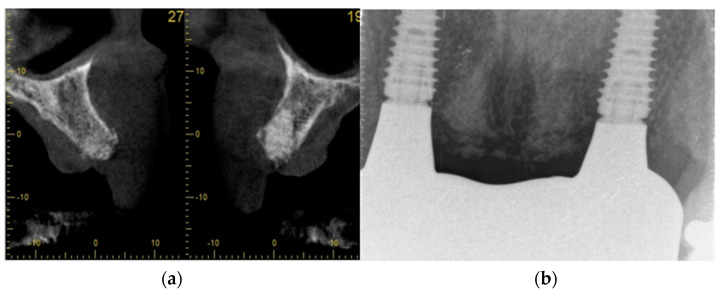
(**a**) shows sagittal view of a CBCT at UR2 and UL2 grafted socket sites. (**b**) a control periapical radiograph showing the stability of marginal bone support around the two Megagen Anyridge^®^ implants in function.

Figure 5a,b is a radiograph illustrating dense radiopacity and contour preservation of the extraction socket 8 weeks after ARP.

Although the implant: crown ratio appears to be unfavorable in this radiograph, the internationally recognized restorative guidelines were used in the selection of implant lengths.

### 3.2. Case Study 2

**Figure 6:**
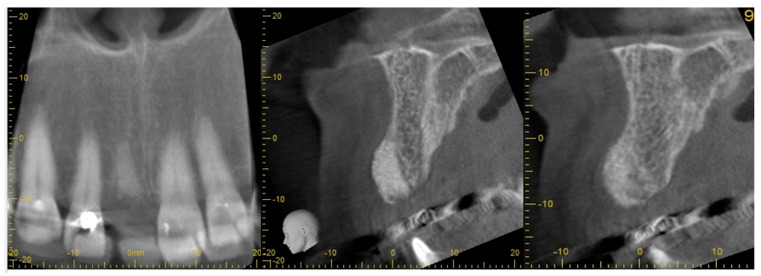
Extraction of UR1 and ARP using BioOss^®^ and A-PRF. A CBCT was taken 8 weeks after ARP showing excellent maintenance of alveolar bone contour with radiologically dense bone.

**Figure 7:**
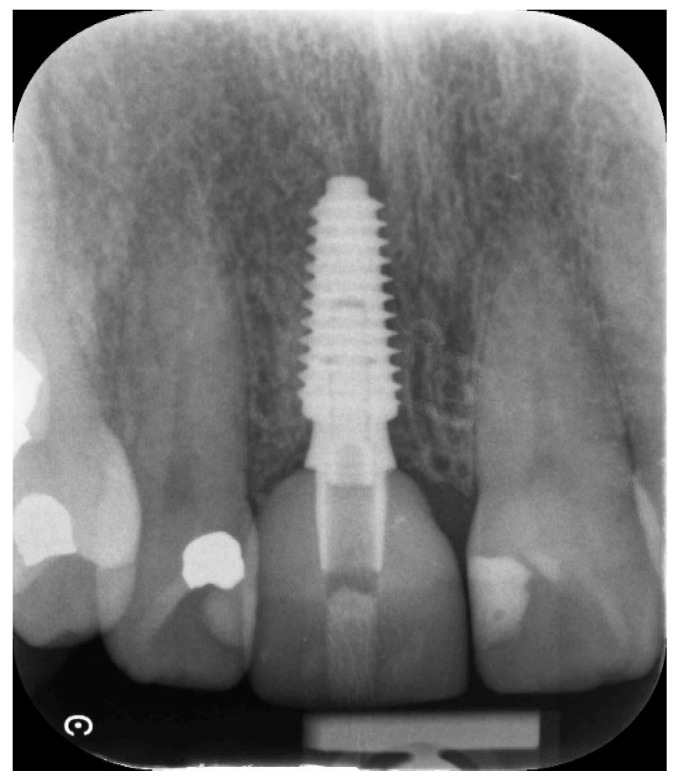
A Megagen Anyridge^®^ implant was placed at 8 weeks after ARP. The implant bed was soaked with an i-PRF solution using a disposable syringe just prior to the insertion of the implants. The periapical radiograph shows good implant integration and maintenance of the newly generated bone within the grafted extraction site at UR1 at a follow-up appointment.

This case presented with a root fracture at UR1. The patient demographic and treatment details are summarized in Table 4. This case was assessed for suitability for either immediate tooth placement or ARP according to published criteria. After full consideration and given the aesthetic expectations, tooth extraction and ARP followed by delayed implant placement option was chosen. UR1 was removed using a minimally traumatic ARP protocol (Ucer and Khan, 2023) [21] with BioOss^®^ and A-PRF. Eight weeks post-ARP, a CBCT scan revealed satisfactory ridge preservation of the alveolar ridge contour with evidence of radiographically dense bone incorporation within the extraction socket. A Megagen Anyridge^®^ implant was inserted 8 weeks post-ARP using the Megagen Anyridge^®^ surgical protocol. Before the implant was inserted, the implant bed was soaked with an i-PRF solution using a single-use syringe. Radiographs indicated successful implant integration and preservation of the newly formed bone at the UR1 extraction site during a subsequent follow-up (Figure 6 and Figure 7 and Table 4).

The results of the two case studies presented in this paper were evaluated using specific primary and secondary outcome measures summarized in Table 4. The primary objective was to assess socket healing and implant integration radiographically and clinically after ARP with PRF.

## 4. Discussion

Alveolar ridge preservation (ARP), also referred to as socket preservation, is a technique used to prevent dimensional changes in an extraction socket in order to facilitate staged implant placement at a later date. This approach is particularly beneficial in cases of thin gingival biotype or extraction socket wall thickness of less than 2 mm in the aesthetic zone. It is also highly recommended when the extraction socket morphology is not suitable to allow immediate implant placement with sufficient primary stability.

Several studies have reported better alveolar crest preservation when graft materials are used in fresh extraction sites [23,24,25,26,27,28,29,30,31,32,33,34,35]. For instance, a randomized controlled trial by Sisti et al. in 2012 showed near-complete vertical and horizontal maintenance of the grafted volume with flapless socket augmentation (ARP) [36]. They also demonstrated that particulate socket grafts without barrier membranes minimized alveolar crest resorption and promoted better horizontal regeneration of the deficient buccal bone wall compared to non-grafted sites. In an SR, Mardas et al. in 2015 concluded that ARP procedures could reduce the need for additional grafting procedures during staged implant placement compared to unassisted socket healing [37]. Nevertheless, they reported the success rate and marginal bone levels of implants placed in alveolar ridges following ARP were found to be comparable to those placed in untreated sockets. Current authors, Ucer and Khan (2023) published a review of the rationale for ARP with platelet-rich fibrin (PRF), which aligns with the findings of Sisti et al. [36] and Mardas et al. [37].

The ideal graft material should be osteoinductive, promoting new bone formation, such as autologous grafts, which remain the gold standard in bone reconstructive surgery. However, the use of autologous bone in socket augmentation is not always practical due to disadvantages, including the need for a donor site. Alternative biomaterials are typically osteoconductive which act as space-maintaining scaffolds. These include allogeneic, xenogeneic, and synthetic derivatives. The main drawback of using scaffold biomaterials is their slow remodeling and consolidation time, which delays implant placement for several months. To enhance new bone formation, biologically active molecules like bone morphogenetic proteins (BMPs) can be added to osteoconductive scaffolds. In the two case studies presented in this paper, implants were placed within 8 weeks after ARP in conjunction with second-generation PRF products (i-PRF). Implants were loaded after a reduced integration period of 6 weeks. Consistent with previous studies, the results demonstrated excellent alveolar ridge preservation after tooth extractions in the anterior maxilla. This allowed successful placement, integration, and early loading of implants without the need for secondary grafting at the time of implant placement. Furthermore, there was no detectable crestal bone loss during the first 12 months of follow-up. These results are consistent with reports in the current literature. Although the optimal timing for implant placement after ARP is currently unknown, there is a consensus that implant placement should be deferred for a period of at least 6 months to allow for the slow incorporation of graft materials, especially xenografts. This view was supported by a recent umbrella review of randomized controlled clinical trials, analyzing the outcomes of flapless socket grafting, which found less than 45% vital bone in ARP grafted with xenografts and allografts after a healing period of 12 weeks [26,27]. 

Platelets and leukocytes are the main cells that are responsible for the biological activity of PRF (Pavlovic et al. 2021, Quirynen and Pinto, 2022) [3,11]. Activated platelet concentrate (PRF) initiates an immune cell response via anti-inflammatory and pro-inflammatory processes that include white blood cells (neutrophils, monocytes, and lymphocytes). The fibrin network acts as a reservoir for platelet-derived growth factors (GFs) (Table 1) involved in all stages of wound healing including angiogenesis, cell recruitment, cell differentiation, mineralization, and tissue regeneration [24,25,26,27,28]. In addition to GFs, PRF is also enriched with leukocytes and immune cytokines (e.g., interleukin) which are the main drivers of bone and soft-tissue regeneration [25].

Leukocytes regulate cell proliferation and differentiation and are essential during tissue injury, inflammatory proliferation, tissue remodeling, and maturation. Neutrophils remove bacteria and necrotic tissue during the inflammatory phase and produce inflammatory cytokines and GFs which are essential for early wound healing. 

Monocytes, evolve into macrophages and are pivotal for tissue healing, angiogenesis, mesenchymal stem cell activation, and tissue regeneration including osteogenesis. PRF matrix has also been shown to exhibit a strong antibacterial capacity to most oral pathogens [26]. Both liquid and solid matrices of PRF release growth factors up to 10 days after reaching a peak at day 7 [38,39]. Protocols for producing PRF variants are shown in Table 1.

Alissa et al. (2010), reported a denser trabecular pattern and less pain sensation along with better soft-tissue healing when platelet rich plasma was used for ridge augmentation. Anitua et al. 2015 [28] reported that platelet rich growth factors (PRGF) yielded good outcomes such as regenerated socket volume, bone density, and soft-tissue healing along with the percentage of new bone formation in mandibular extraction sockets. However, the results obtained with PRGF, which is depleted of leucocytes, cannot be translated to PRF due to their different properties. Production of PRGF requires the addition of a CaCl solution followed by heat treatment. Others have reported beneficial ARP results with increased crestal ridge width and a higher percentage of vital bone [29,30]. Wu et al. 2012 found that PRF stimulates PDLF proliferation as well as osteoblast and gingival fibroblast proliferation by 1.28-fold collectively (*p* < 0.05), thus, increasing post-operative bone regeneration. PRF has also been shown to reduce the overall wound-healing time [31].

There is compelling biological evidence supporting the use of PRF as a bioactive material for enhancing tissue regeneration and wound repair. Numerous biochemical studies have demonstrated that PRF contains a significant concentration of biologically active matrix proteins and growth factors (GF), which are released gradually due to the three-dimensional architecture of glycoproteins in the fibrin clot (refer to Table 1). Additionally, PRF is believed to play a role in the osteoimmune response, which arises from the interaction between bone and the immune system. GFs are expressed in both PRF matrices and contribute to the enhancement of angiogenesis and early wound healing [32,33].

In an animal study, Yuan et al. (2021) applied PRF with a resorbable gel and found it effective in ridge preservation by promoting blood clotting, angiogenesis, and osteogenesis [25]. Hauser et al. used PRF preparations in 168 post-extraction sockets in 50 cardiac surgery patients without modifying their anticoagulant therapy (mean international normalized ratio = 3.16 ± 0.39). In all cases, there were no reports of alveolitis or painful events, and wound closure was complete at the time of suture removal one week after surgery. They proposed using the PRF protocol as a reliable therapeutic option to prevent significant bleeding after dental extractions without suspending continuous oral anticoagulant therapy in heart surgery patients [34].

In a case study, Chenchev et al. (2017) reported the benefits of using A-PRF and i-PRF in conjunction with a bone substitute in ARP before implant placement [27]. In a rare split-mouth randomized controlled clinical trial, Temmerman et al. (2016) investigated the effect of using a preparation of platelet-rich fibrin, as a socket-filling material, on ridge preservation. In twenty-two patients in need of single bilateral and closely symmetrical tooth extractions in the maxilla or mandible, they reported significant differences (*p* < 0.005) in total width reduction between test (−22.84%) and control sites (−51.92%) at 1 mm below the crest level. Significant differences were also found for socket fill between the test (94.7%) and control sites (63.3%). They concluded that the use of PRF was effective in preserving horizontal and vertical ridge dimensions 3 months after tooth extraction, with clear benefits [36].

In another split-mouth RCT, Castro et al. (2021) [30] studied the effect of different platelet-rich fibrin preparations (A-PRF+ and LPRF) and reported that while the mean horizontal and vertical changes at 1-mm below the crest (buccal and palatal side) were similar for the test and control sites (*p* > 0.05), both PRF matrices showed radiographically significant superiority for socket fill (L-PRF (85.2%) and A-PRF+ (83.8%) compared with the controls (67.9%) [30,31,32,33,34,35,36]. A systematic review by Al-Maawi (2021) [32] of 20 RCTs and controlled studies showed that PRF was effective in reducing post-operative pain, accelerating soft-tissue healing, and preventing dimensional bone loss, especially in the early period of 2–3 months. Dimensional bone loss was significantly lower in the PRF group compared with unaided wound healing after 8–15 weeks but not after 6 months. Socket fill was significantly higher in the PRF group compared with spontaneous wound healing. The authors concluded that based on the analyzed studies, PRF was most effective in the early healing period of 2–3 months after tooth extraction and allowing a longer healing period may not provide any additional benefits. However, the authors found heterogeneity between the included studies and assessed a relatively high risk of bias in the blinding of participants and personnel, as well as the blinding of outcome assessment [32,33,34,35,36].

In a multi-arm parallel randomized controlled clinical trial, Clark et al. (2018) evaluated the efficacy of platelet-rich fibrin (A-PRF) alone or mixed with freeze-dried bone allograft (FDBA) in improving vital bone formation and alveolar dimensional stability during ARP in fresh extraction sites [33]. In 40 patients, non-molar extraction sites were randomized into one of four ridge preservation approaches: A-PRF, A-PRF+FDBA, FDBA, or a blood clot. They concluded that A-PRF alone or augmented with FDBA was an effective biomaterial for ridge preservation [33]. This finding aligns with recommendations by Miron et al. (2021) to use PRF alone in intact premolar and molar sites to prevent dimensional changes, reduce pain and infection, and enhance wound healing. However, when the buccal wall of an extraction socket is missing, the use of a bone graft mixed with PRF is advisable.

Bernnardo et al. (2023) reported that PRF loaded with antibiotics allowed the release of effective concentrations of antimicrobial drugs and could potentially reduce the risk of post-operative infection after oral surgery. However, further studies are needed to confirm the efficacy of PRF as a topical antibiotic delivery tool [33,34].

As demonstrated in the two case studies presented in this paper, combining platelet-rich fibrin (PRF) with alveolar ridge preservation (ARP) may facilitate earlier patient rehabilitation through improved wound healing and bone regeneration. Emerging biological and clinical evidence suggests that second-generation PRF products can significantly enhance biological healing processes when used as an adjunct during ARP, potentially shortening the overall duration of staged implant treatment following tooth extractions. 

Additionally, coating implant surfaces or soaking the implant bed with liquid PRF may further enhance the osseointegration process in augmented or native implant sites. This perspective is supported by laboratory and in-vivo randomized controlled trials (RCTs) conducted by Oncu and Alaaddinoglu et al., Oncu, Erbeyoglu et al., and Quirynen and Pinto, 2022 [11,38,39]. These studies reported higher implant stability quotient values and significantly reduced bone loss from post-implantation bone remodeling when PRF was applied during implant placement. 

Nevertheless, it must be pointed out that current lack of comparative RCTs makes it difficult to determine the clinical significance of different PRF preparations or the ideal timing of interventions during ARP. A literature review on this subject revealed inconsistencies in study methodologies and a lack of uniform outcome measures (e.g., implant success/survival, marginal bone loss, and implant stability), preventing comprehensive scientific analysis. As a result, the evidence for the clinical efficiency of PRF and its variants in bone regeneration in dental implantology has not been fully established [36,37,38,39]. Further research is urgently needed to establish the optimal ARP and PRF protocols with standardized methods and outcome measures.

While existing evidence and practical applications show PRF as a viable tool to expedite wound healing and tissue regeneration, especially in dental applications such as ARP, comprehensive research is requisite to define standardized protocols and explore optimal applications of PRF in tissue regeneration and healing.

## 5. Conclusions

In conclusion, emerging evidence supports the biological and clinical advantages of utilizing PRF in conjunction with ARP to enhance all phases of wound healing and tissue regeneration. The case studies presented in this paper provide further evidence that this approach has the potential to allow for earlier rehabilitation of patients and reduce the overall duration of staged implant treatment after tooth extractions when PRF is used as an adjunct during ARP. Additionally, good primary stability was achieved and there was no need for secondary grafting when implants were placed 8 weeks after ARP. While further research is required to establish optimal protocols and elucidate the bio-enhancement properties of PRF, the existing evidence strongly indicates its promising role in dental implantology and tissue regeneration.

## Figures and Tables

**Table 1:** Protocols for producing different formulations (solid and liquid) of PRF [12]. Solid forms of PRF include Advanced PRF preparations (A-PRF, A-PRF+) and leucocyte rich PRF (L-PRF). Liquid PRF formulations are injectable PRF (i-PRF) and concentrated PRF (C-PRF).

PRF Preparation	Tube	RCF (g)	Time (min)	Speed (rpm)	Evidence
Solid Matrix					
L-PRF	Glass or Silica coated	408	12	2700	Choukroun, 2001 [12]
A-PRF	Glass or Silica coated	194	14	1500	Ghanaati et al., 2014 [7]
A-PRF+	Glass or Silica coated	145	8	1300	Fujioka-Kobayashi et al., 2016 [13]
Liquid matrix					
i-PRF	Plastic (PET)	60	3	700	Miron et al., 2017 [8]
C-PRF	Plastic (PET)	408	12	2700	Miron et al., 2020 [10]

**Table 2:** Conversion table for calculating g-force from rpm and radius arm of the centrifuge device (Adapted from Sigma Aldrich).

Radius (cm)	4	5	6	7	8	9	10	11	12	13
Speed (rpm)										
1000	45	56	67	78	89	101	112	123	134	145
1500	101	126	151	176	201	226	252	277	302	327
2000	179	224	268	313	358	402	447	492	537	581
2500	280	349	419	489	559	629	699	769	839	908
3000	402	503	604	704	805	906	1006	1107	1207	1308

**Table 3:** Platelet-Derived Growth Factors found in PRF that have a direct role in early wound healing. These factors and cytokines released from processed platelets are involved in mesenchymal cell recruitment, cell differentiation and activation, angiogenesis, and wound healing processes.

Growth Factors	Functions
Transforming Growth Factor (TGF)	Growth of endothelial vascular cells, cell recruitment, and proliferation in wound healing. Inhibits osteoclast formation and bone resorption. Stimulates fibronectin and collagen production.
Epidermal Growth Factor (EGF)	Promotion of mesenchymal cell proliferation and differentiation, epithelial cell growth, and angiogenesis
Vascular Endothelial Growth Factor (VEGF)	Restores oxygen supply to the injured tissue. Promotes repair and growth of vascular endothelial cells, and angiogenesis
Platelet-Derived Growth Factors (PDGF)	Cell growth, proliferation of smooth muscle cells within vascular tissue, angiogenesis, and collagen production Provokes proliferation of mesenchymal cell lineage, and enables macrophage chemotaxis
Insulin-like Growth Factor (IGF)	Cell proliferation, cell-to-cell communications, stimulates chemotaxis and activation of osteoblasts and bone formation, and induces mitogenesis of mesenchymal cells
Fibroblast Growth Factor (FGF)	Tissue repair, cell growth, hyaluronic acid and collagen production

**Table 4:** Outcomes of case studies presented in our study.

Case No:	Age, Gender, Medical History	ARP Technique	AR Volume	BQ after ARP	PS	BQ at Implant Placement	Secondary Grafting at Implant Placement	Time Since ARP	Early Loading	CBS
Case 1	60, male, prediabetes, non-smoker with no medication	open ARP	Full contour preserved	excellent	high	D2-3	None	8 weeks	6 weeks	No crestal bone loss
Case 2 (implant 1)	55, female, hay fever, non-smoker	open ARP	Full contour preserved	excellent	high	D2-4	None	9 weeks	7 weeks	No crestal bone loss
Case 2 (implant 2)	55, female, hay fever, non-smoker	open ARP	Full contour preserved	excellent	high	D2-4	None	9 weeks	8 weeks	No crestal bone loss

ARP: alveolar ridge preservation; AR volume: Full contour preservation; BQ after ARP: (radiological bone quality) excellent/good/poor; PS: Primary implant stability at placement: High (>40 Ncm)/medium (30 to 40 Ncm)/Low (less than 30 Ncm); Surgery BQ: bone density/quality at implant surgery D1; D2; D3; D4; Secondary grafting: Need for additional grafting at the time of implant placement; ARP: Time since ARP (weeks) at the time of implant placements; Early loading: Time since implant placement (weeks); CBS: Radiological signs of crestal bone stability.

## Data Availability

Not applicable.
